# Impact of COVID-19 on the changing pattern of human orthopneumovirus (respiratory syncytial virus) infection in Iran

**DOI:** 10.1186/s12879-023-08588-z

**Published:** 2023-09-11

**Authors:** Jila Yavarian, Somayeh Shatizadeh Malekshahi, Marziyeh Faraji-Zonouz, Shirin Kalantari, Sevrin Zadheidar, Sara Saghafi, Faezeh Tarpour, Arash Letafati, Akram sadat Ahmadi, Nazanin-Zahra Shafiei-Jandaghi, Talat Mokhtari-Azad

**Affiliations:** 1 Virology Department, School of Public Health, Tehran University of Medical Sciences, Tehran, Iran; 2 Research Center for Antibiotic Stewardship & Antimicrobial Resistance, Tehran University of Medical Sciences, Tehran, Iran; 3 Department of Virology, Faculty of Medical Sciences, Tarbiat Modares University, Tehran, Iran

**Keywords:** Respiratory syncytial virus, Human orthopneumovirus, COVID-19, Iran

## Abstract

**Background:**

Human orthopneumovirus (HOPV) or respiratory syncytial virus (RSV) is one of the important causes of acute respiratory infections (ARIs) during the cold months of the year worldwide. Many countries have reported an absence of ARIs due to HOPV during the winter of 2020–2021 associated with preventive measures to reduce the spread of SARS-CoV2. However, with the reduction of COVID-19 public health restrictions and the absence of immunity in the community due to the lack of exposure in the previous season, many countries had a delayed HOPV outbreak. Here we reported the impact of COVID-19 on the changing pattern of HOPV infection in Iran.

**Methods:**

Throat and nasopharyngeal swab samples were collected from patients (children and adults) with ARIs and sent to the Iran National Influenza Center. After RNA extraction, Real time RT-PCR was performed for HOPV detection.

**Results:**

In 260 samples collected from patients with ARIs in three different groups, which included children in March 2021, pilgrims in July 2022, and outpatients during November and December 2022, no HOPV was detected in any group.

**Conclusions:**

The lack of HOPV activity in Iran during the winter of 2020–2021 and then the resurgence in spring 2022 and again the absence of activity in summer and autumn 2022 was extraordinary in the HOPV epidemiology, and probably due to the implementation of public health non-pharmaceutical interventions to reduce the spread of SARS-CoV2. Although it is not possible to keep such restrictions, similar methods can be taken to control outbreaks caused by respiratory viruses.

## Introduction

Human orthopneumovirus (HOPV) or human respiratory syncytial virus (HRSV) belongs to the genus *Orthopneumovirus* within the family *Pneumoviridae* and is able to cause severe disease in different groups, including premature newborns, children, the elderly, and immunocompromised people [1]. The human orthopneumovirus genome consists of a 15.2 kb negative-sense single-stranded RNA containing 10 genes that encode 11 proteins. Based on the variations found in the G gene sequence its strains can be divided into two subtypes A and B [2]. Until 2019, HOPV had predicted seasonality patterns with annual epidemics in winter months, especially in the northern hemisphere [3]. In Iran, HOPV activity peaks during February and March every year.

The emergence of severe acute respiratory syndrome coronavirus 2 (SARS-CoV2) and the subsequent coronavirus disease 2019 (COVID-19) pandemic caused considerable changes in the circulation of respiratory viruses with a dramatic absence of HOPV and influenza activity [4].

Human orthopneumovirus is transmitted via respiratory droplets and indirect contact with contaminated surfaces like SARS-CoV2. During the COVID-19 pandemic, preventive measures were widely implemented to decrease the spread of SARS-CoV2, including physical distancing, facemasks, stay-at-home orders, work, schools, and border closures, and handwashing. All these measures have the potential to prevent the transmission of HOPV and influenza [5].

As public health non-pharmaceutical mitigation measures were reduced during the spring of 2021, an extraordinary resurgence of HOPV cases occurred worldwide [6]. The reasons for this unusual resurgence of RSV cases were uncertain, but it might be due to the lack of HOPV immunity because of the extended absence of virus circulation in the community [7]. During the COVID-19 pandemic, study about the effect of the pandemic on the changing pattern of other respiratory viruses and subsequent resurgence has increased worldwide.

Here, we studied HOPV circulation during the COVID-19 pandemic in different intervals and performed laboratory test for HPOV detection to show the impact of COVID-19 on the circulation pattern of HOPV in Iran.

## Methods

### Study population

Throat and nasopharyngeal swab samples were collected from different groups with acute respiratory infections (ARIs) for HOPV detection and the samples were sent to the Iranian National Influenza Center (NIC), Virology Department, School of Public Health, Tehran University of Medical Sciences. The groups were: children under 5 years of age who attended Children Medical Center, Tehran, Iran in March 2021, returning pilgrims from Mecca in July 2022, and outpatients from hospitals in different cities in autumn 2022. We obtained ethical clearance (including permission to study the children) from Tehran University of Medical Sciences Medical Ethics, ref no: IR.TUMS.REC.1399.306. Written informed consent was obtained from all patients as the samples were taken for influenza surveillance via the Ministry of Health and Education.

### Nucleic acid extraction and real-time PCR

Ribonucleic acid (RNA) was extracted from all specimens by High Pure Viral Nucleic Acid kit (Roche, Germany) according to the manufacturer’s instructions. Detection of HOPV was performed using one-step real-time RT-PCR with the Biotech Rabbit kit (Japan). The nucleocapsid gene was selected for the amplification of HOPV RNA using RSV N forward primer: 5’-GCTAAAAGAAATGGGAGAGG-3’; RSV N reverse primer: 5’-TAATCACGGCTGTAAGAC C-3’ and RSV N probe: 5’-FAM AGCTCCAGAATACAGGCATGACTC-TAMRA-3’ [8]. RNA (5 µl) was added to a 20 µl PCR mixture containing 12.5 µl of mastermix, 1 µl of enzymemix, 1 µl of each primer (10 pmol), 0.5 µl probe (10 pmol) and, 4 µl ddH2O. The reaction consisted of 20 min at 50 °C and 2 min at 95 °C, followed by 40 cycles of amplification, including 30s at 95 °C and 30s at 54 °C.

## Results

In total, 260 samples were taken from patients in all three groups but HOPV was not detected in any sample.

The first studied group was the children less than 5 years old hospitalized with ARIs at Children Medical Center in March 2021. Of 122 children, 69 (56.5%) were male, and 53 (43.5%) were female. We divided patients into two age groups: less than 1 year old and 1–5 years old. 37 (30.5%) children were younger than 1 year old and 85 (69.5%) were 1–5 years old. The most common clinical manifestations of patients were cough, nausea, diarrhea, and fever. Among 122 patients, no HOPV was detected.

The second studied group was the pilgrims who returned from Mecca after the pilgrimage, which was held after two years of closure because of the COVID-19 pandemic. All returning pilgrims to Tehran International Airport in July 2022, were screened for respiratory symptoms and signs, of whom 26 had at least cough, sore throat, and fever. The age range was 27–73 with a median of 53 years. The male to female ratio was 1. Human orthopneumovirus was not detected in these 26 pilgrims.

The third studied group was the adult outpatients with ARIs from different cities in Iran. Their samples were sent to NIC for screening of different respiratory viruses from November to December 2022. In all 112 samples, 62 (55.4%) were female and 50 (44.5%) were male. Human orthopneumovirus was not detected in these samples. It should be mentioned that public health interventions undertaken during the COVID-19 pandemic have led to a low circulation of HPOV cases.

## Discussion

Before the COVID-19 pandemic, HOPV was an important cause of ARIs in children (under 5) and the elderly and was a significant burden on the public health system of Iran. Two systematic reviews about the prevalence of HOPV in Iran have been conducted; one covered from the 1996–2013 period and the other reviewed from 1996 to 2016. They showed the presence of HOPV 18.7% and 18% respectively during the cold months of the year (November-March) [9, 10]. Between January to March 2017, HOPV was detected in 2.75% of military trainees with ARIs in Tehran, Iran [11]. Another study in Tehran, Iran, between January and March 2018, and December 2018 to May 2019 among hospitalized or outpatient children younger than 2 years of age showed a prevalence of 35.92% HOPV with a peak in March followed by February [12].

The SARS-CoV2 pandemic has significantly changed the epidemiology of seasonal respiratory infections worldwide, for instance, HOPV epidemics during the cold months have disappeared during 2020 and 2021 [13]. In Iran, a study between March and May 2021 in children younger than 5 years old showed the absence of HOPV circulation during the COVID-19 pandemic which indicated the impact of a pandemic on HOPV circulation in Iran as shown in different studies worldwide [14]. In current study, HOPV was not detected in 122 hospitalized children in March 2021, 26 pilgrims in July 2022, and 112 outpatients in autumn 2022. Our findings were comparable with the reports from other countries that have reported decreased HOPV circulation in the first winter following the COVID-19 pandemic. For example, an analysis of HOPV activity in United States during 2020–2021, reported by the Centers for Disease Control and Prevention (CDC) revealed that circulation of HOPV decreased in early 2020 [15]. Similarly, in Australia, HOPV activity decreased 98% during the winter of 2020 [16]. Also findings were reported from Japan where the number of HOPV cases during January–December 2020 were reduced by almost 85% compared to 2014–2019 [17]. A study in Korea reported a 19% reduction in positive rates of HOPV during the COVID-19 pandemic [18]. A Chinese study showed the absence of HOPV circulation in 2020 [19]. A research study from Italy reported a significant reduction in HOPV circulation following the appearance of SARS-CoV2 in 2020–2021 [20, 21]. Another study from Finland reported that HOPV circulation had the pattern of previous years in the first 12 weeks of 2020 but then reduced, because of nationwide restrictions [15].

As mentioned above, more than one year after the COVID-19 pandemic, the circulation of HOPV was reduced in different countries. Potentially such reduction could cause a general increase in susceptibility to infections because of the possible reduction in immunity in society, which might lead to large epidemics of HOPV. Surveillance data from several countries have reported delayed HOPV outbreaks resulting from easing the restrictions and restarting social activities [22]. In Australia, the inter-seasonal resurgence of HOPV in children after the relaxation of COVID-19 restriction measures was reported [16]. A study in Israel showed a temporal move of the HOPV epidemic during 2021 among hospitalized children in Ashod, with a lack of HOPV circulation during the autumn/winter of 2020/21, and a delayed resurgence of cases from May to June 2021 [23]. Surveillance data from different countries including the Netherlands, France, Iceland, and Japan have shown a delay in the onset of the HOPV epidemic after easing the COVID-19 pandemic measures [22, 24]. In a study in Iran from December 2020 to March 2021 among 197 hospitalized patients suspected of COVID-19 with the age range of 45–68 years HOPV was detected in four (2.03%) patients [25]. In April 2022, another study in Iran, reported a delayed resurgence of HOPV in a small population of children in the southwest of Iran, after the lack of HOPV circulation from the beginning of the COVID-19 pandemic [6]. As shown here the change in HOPV seasonality reveals similar findings reported elsewhere. One of the limitations of our study is that the period and number of samples taken were not sufficient to provide more complete and precise epidemiological information on RSV during COVID-19 in Iran.

There have been limited data about the prevalence of HOPV in adults during the COVID-19 pandemic because most studies focused on children. As stated our study included a good number of adults to understand the impact of the COVID-19 pandemic on HOPV activity. As noted above HOPV was not detected in all three groups, emphasizing that the pandemic has impacted the circulation of HOPV in all populations. Our study confirms the change in HOPV seasonality indicated in other studies worldwide and we reiterate the significance of control measures, behavioral changes, and surveillance systems during a pandemic.

## Data Availability

All data generated or analyzed during this study are included in this article.

## List of abbreviations

RSV: Respiratory syncytial virus
SARS-CoV2: Severe acute respiratory syndrome coronavirus 2
COVID-19: Coronavirus disease 2019
NIC: National Influenza Center
ARIs: Acute respiratory infections
RNA: Ribonucleic acid
HOPV: Human orthopneumovirus
